# Feasibility study of immersive virtual prism adaptation therapy with depth-sensing camera using functional near-infrared spectroscopy in healthy adults

**DOI:** 10.1038/s41598-022-04771-5

**Published:** 2022-01-14

**Authors:** Sungmin Cho, Won Kee Chang, Jihong Park, Seung Hyun Lee, Jongseung Lee, Cheol E. Han, Nam-Jong Paik, Won-Seok Kim

**Affiliations:** 1 Department of Rehabilitation Medicine, Seoul National University College of Medicine, Seoul National University Bundang Hospital, 82, Gumi-ro 173 Beon-gil, Bundang-gu, Seongnam, Gyeonggi-do 13620 Republic of Korea; 2 Global Health Technology Research Center, College of Health Science, Korea University, Seoul, Republic of Korea; 3 Department of Electronics and Information Engineering, Korea University, Sejong, Republic of Korea; 4 Interdisciplinary Graduate Program for Artificial Intelligence Smart Convergence Technology, Korea University, Sejong, Republic of Korea

**Keywords:** Neuroscience, Neurology

## Abstract

Prism Adaptation (PA) is used to alleviate spatial neglect. We combined immersive virtual reality with a depth-sensing camera to develop virtual prism adaptation therapy (VPAT), which block external visual cues and easily quantify and monitor errors than conventional PA. We conducted a feasibility study to investigate whether VPAT can induce behavioral adaptations by measuring after-effect and identifying which cortical areas were most significantly activated during VPAT using functional near-infrared spectroscopy (fNIRS). Fourteen healthy subjects participated in this study. The experiment consisted of four sequential phases (pre-VPAT, VPAT-10°, VPAT-20°, and post-VPAT). To compare the most significantly activated cortical areas during pointing in different phases against pointing during the pre-VPAT phase, we analyzed changes in oxyhemoglobin concentration using fNIRS during pointing. The pointing errors of the virtual hand deviated to the right-side during early pointing blocks in the VPAT-10° and VPAT-20° phases. There was a left-side deviation of the real hand to the target in the post-VPAT phase, demonstrating after-effect. The most significantly activated channels during pointing tasks were located in the right hemisphere, and possible corresponding cortical areas included the dorsolateral prefrontal cortex and frontal eye field. In conclusion, VPAT may induce behavioral adaptation with modulation of the dorsal attentional network.

## Introduction

Stroke is the leading cause of acquired disability worldwide and causes various kinds of impairments^1^. Although motor impairment including hemiplegia is most common^2^, recovery from non-motor impairments is also important and is targeted for rehabilitation in patients with stroke^3^. Unilateral spatial neglect (USN) (a deficit in attention to and awareness of the contralesional side of space), one such non-motor impairment, is common and persists usually after right hemisphere damage^4,5^. Because USN is associated with poor functional recovery and lower social return, appropriate rehabilitative intervention has to be provided^6,7^.

Among various rehabilitation approaches, including both top-down approaches such as visual scanning, and bottom-up approaches including prism adaptation (PA), caloric vestibular stimulation and neck vibration^8,9^, PA is one of the most supported modality able to ameliorate USN^10^.

The conventional PA setting consists of prism glasses with a displacement of 10° or 20°^11^, a visual target and masking devices to conceal the hand trajectory in proximal part and allow the exposure of hand only near the target. Participants make pointing errors due to optical shift in the early phase but gradually correct the errors and re-gain baseline pointing accuracy in the late phase of prism exposure. After-effect is measured as the degree of error in pointing after the prism glasses are removed, using open-loop pointing defined as pointing tasks without visual feedback of the participants’ movement^12^, to measure the total visuomotor shift^13^.

The action of PA has been widely explained with two key neural mechanisms; recalibration and realignment^14,15^. Recalibration is a cognitive compensatory response to modify motor command during object reaching and is an immediate reaction to reduce the terminal error caused by the prism deviation. Realignment refers to a slow, automatic process which reorganizes displaced coordinate systems by prisms and is mainly responsible for the after-effect of PA^12^. These two processes are known to operate and exist independently of each other^13^. Panico et al. have reviewed the underlying cortical activities and structures related to PA and have reported that the parietal cortex and cerebellum are pivotal regions in PA, however, the functional role of these areas in regard to recalibration and realignment remains unclear^16^.

Implementation of PA to ameliorate USN in stroke patients has been reported in previous studies, although the effect of PA remains inconclusive. Many studies have reported beneficial effects of PA; Serino et al.^17,18^, Mizuno et al.^19^, and Shiraishi et al.^20^ have reported beneficial effects of PA on USN. Franssinetti et al.^21^ and Rusconi and Carelli^22^ demonstrated the effect of PA sustained in long-term. In contrast, Ten Brink et al.^23^ and Nys et al.^24^ have reported no significant effects of PA on USN. This variation could be due to differences in PA configurations and outcomes being measured. Negative results have been also published leaving the effect of PA inconclusive.

Currently PA is underapplied in clinical practice due to several issues regarding conventional PA^11,25^. First, the setting for masking the hand trajectory and visual targets is burdensome and requires a certain amount of space. This often impedes PA application to patients who cannot sit stably or control their head in a sitting position. Second, the exposure session during conventional PA has to be conducted under the supervision of a therapist, which limits its use in the ward or home. Third, PA’s degree of adaptation and after-effect are hard to quantify. If these problems can be solved, PA can become more beneficial to patients with extended PA sessions run in a more effective manner, resulting in long-lasting improvements^26^.

Recently, studies have reported on applying immersive VR in PA. Gammeri et al.^27^ tested a PA system with gradual visual shift of a virtual rod in healthy participants, and demonstrated that the transfer effect was observed in the large shift group. Ramos et al.^28^ assessed the after-effect in VR using two different methods of inducing visual shift (visual rotation and visual skew) and discovered that PA using VR produces larger after-effect than conventional PA. Wilf et al.^29^ determined that PA using VR and passive adaptation training can induce after-effect. Conversely, Bourgeois et al.^30^ found no transfer effect when applying VR PA to neglect patients.

We developed a new PA system, “Virtual Prism Adaptation Therapy (VPAT),” using immersive virtual reality (VR) and a depth-sensing camera to overcome the limitations of conventional PA. VPAT deviates the hand trajectory during pointing through VR. Since VR can change the amount of visual displacement, and selectively show information, our VPAT does not require bulky system for masking the hand trajectory. This new system is easy to use, requires less supervisions, and automatically quantifies the degree of adaptation and after-effect. We designed an experiment with healthy individuals to investigate whether VPAT can induce similar effects to conventional PA. As a first surrogate outcome, the pointing errors from the target during the adaptation and post-adaptation period were employed. The second surrogate outcome was the cortical activation pattern during pointing with or without the prism mode, during the adaptation and post-adaptation periods. Functional near-infrared spectroscopy (fNIRS) was used to investigate possible neural substrates related to VPAT. fNIRS was selected as a neuroimaging modality in our study based on several advantages in task-involved studies: (1) an acceptable temporal sampling rate, (2) suitability for continuous data acquisition over a long period, (3) resistance to motion artifacts, (4) it is unaffected by electromagnetic fields, and (5) low physical burden to the participants compared to other neuroimaging modalities^31,32^.

## Methods

### Participants

Inclusion criteria for this study were healthy individuals aged between 18 and 50 years, right handed—assessed by the Edinburgh handedness inventory^33^, feasibility to wear the Oculus Rift DK2 (Oculus VR, LLC, CA, USA), and the ability to detect objects in immersive VR. Subjects with any history of disease involving the central nervous system such as stroke, traumatic brain injury, or Parkinson’s disease were excluded. All subjects provided written informed consent for participation. Although this was a pilot study, we calculated sample size. Thirteen subjects were needed to detect a 1° pointing bias with standard deviation of 0.9° after VPAT (with β = 0.20 and α = 0.05) based on a previously published study in healthy subjects with PA^34^. Fourteen healthy subjects (seven male, seven female), aged between 22 and 39 (27.8 ± 4.1) years, participated in this study. This study was carried out according to the Declaration of Helsinki and Good Clinical Practice Guidelines, and the protocol was approved by the Seoul National University Bundang Hospital Institutional Review Board. This study protocol and preliminary behavioral results, without fNIRS data in four study subjects, were published previously^35^.

### Concept of the VPAT system

VR systems with head mounted displays provide immersive visual feedback. Hand tracking with a head mounted display eliminates the gap between the virtual hand location and the location of the virtual object in virtual space. This clearly differs from systems using a 2D or 3D display, stylus, and mouse. Synchronization of the coordinate system of VR contents and hand tracking coordination can induce more realistic interactions with virtual objects. It is also possible to intentionally misalign the virtual hand and virtual object during the pointing action. This may be similar to a visual field shifting in the PA paradigm using prism goggles. However, while prism goggles shift all visual fields, our VPAT system only shifts the virtual hand trajectory in the immersive VR.

**Figure 1:**
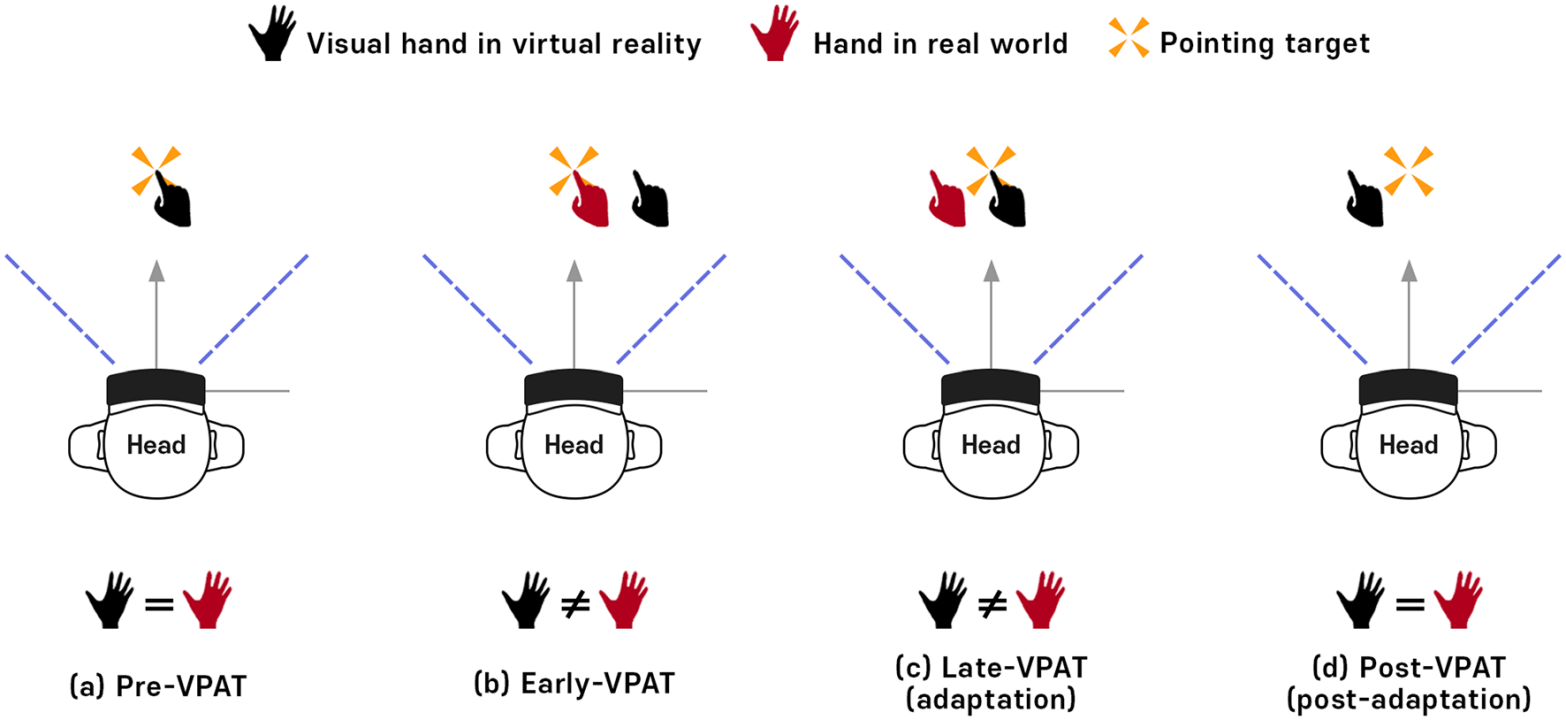
Concept of virtual prism adaptation therapy (VPAT). (**a**) Pre-VPAT: The position of the virtual hand in virtual reality and the real hand are always the same. (**b**) Early VPAT: Virtual hand shifted to the right side from the real hand. Virtual and real hand positions are not the same. (**c**) Late VPAT (adaptation): After adaptation, the virtual hand will reach the target correctly, but the real hand will deviate to the left side from the object. (**d**) Post-VPAT (post-adaptation): Virtual and real hand are in the same position and the real hand still points the left direction from the target.

The virtual hand shift using our VPAT system induces initial pointing errors. In the condition without VPAT mode (no virtual hand shift), the subject directly points the virtual target (Fig. 1a). During the early VPAT period, the subject’s virtual hand is shifted to the right from the VR target (Fig. 1b), which might make the subject perceive the real target location to the left of the initial pointing location. During the late VPAT period, the subject adapts to VPAT mode and the pointing errors of the virtual hand to the target diminish and finally disappear (Fig. 1c). After the VPAT mode is switched off, the subject’s real hand will point to the left of the target (Fig. 1d), if the adaptation effect of VPAT is similar to conventional PA therapy.

### Hardware and software design of the VPAT system

**Figure 2:**
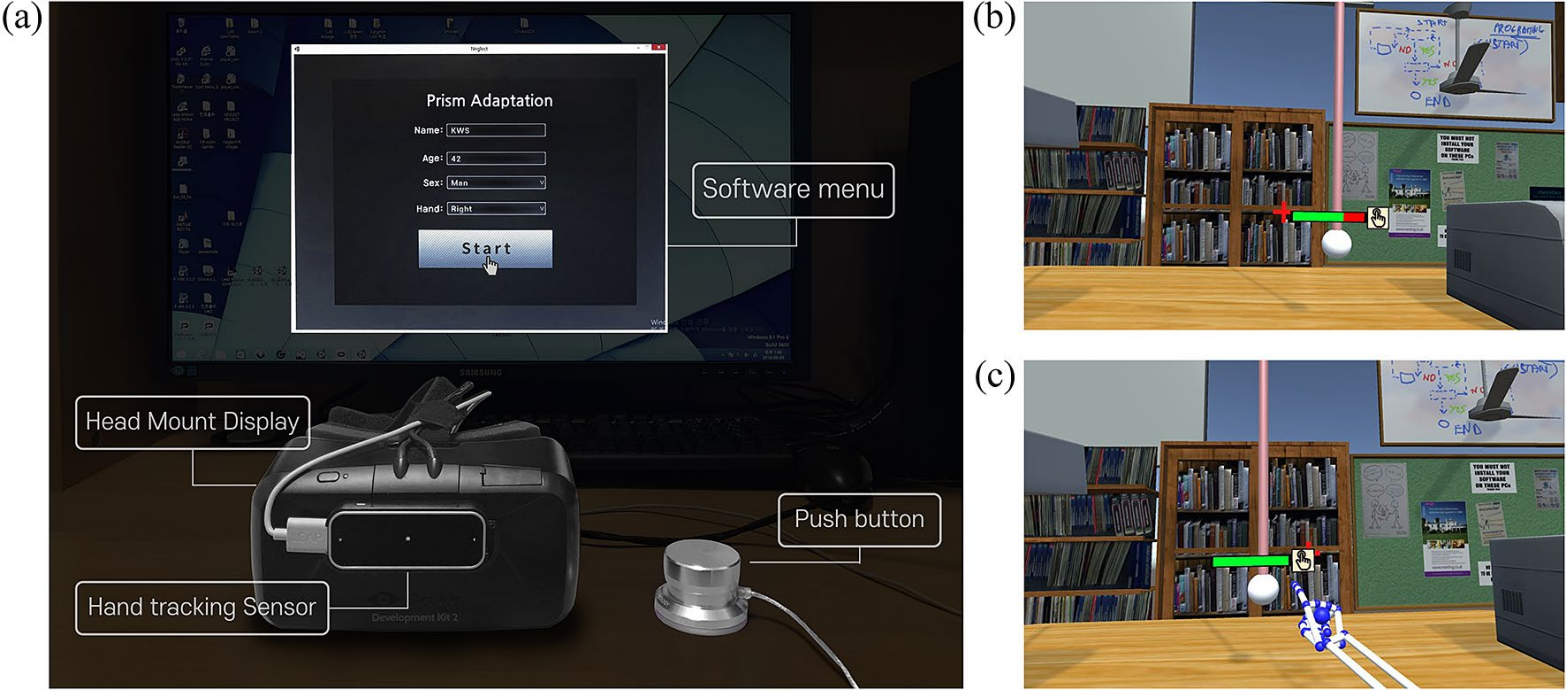
VPAT system configuration. The Oculus rift DK2 was used as a head mount display for VR rendering. Leap motion was used for hand tracking. (**a**) A push button was used during clicking sessions in our experiment, and the software menu was constructed to save user’s information including name, age, gender, and the hand side used for training. (**b**) Environmental design for the experiment. The red cross marked on the bookshelf is used to calibrate the body and center of vision. A white ball is used as the target for pointing. The remaining trial time is indicated by a health bar, and to the right is an icon showing the current task. (**c**) Visual feedback is only given when the index finger is reached into visible area.

The VPAT system uses the Oculus Rift DK2 with Runtime Software version 2.1.2 (Oculus VR, LLC, California, USA, https://developer.oculus.com/downloads/unity/) as a head mounted display (HMD), while the Leap Motion sensor with Orion software version 3.1.3 (Leap Motion, CA, USA, https://developer.leapmotion.com/releases/?category=orion) is mounted on the front of the HMD for hand tracking (Fig. 2a). Before projecting the user’s hands into the virtual space in real time, the hand skeleton dummies detected by the Leap Motion sensor are rotated based on the longitudinal axis for the visual hand shift.

The VR environment was created with Unity3D version 5.4.3 (Unity Technologies, CA, USA, https://unity3d.com). The environment is based on a general living environment and composed of an office with a desk (Fig. 2b). The objects used in the environment were constructed to avoid interfering with the view or interaction with the target. The red cross marked on the bookshelf is used to calibrate the body and center of vision. A white ball is used as the target for pointing. The remaining trial time is indicated by a health bar, and to the right is an icon showing the current task.

**Figure 3:**
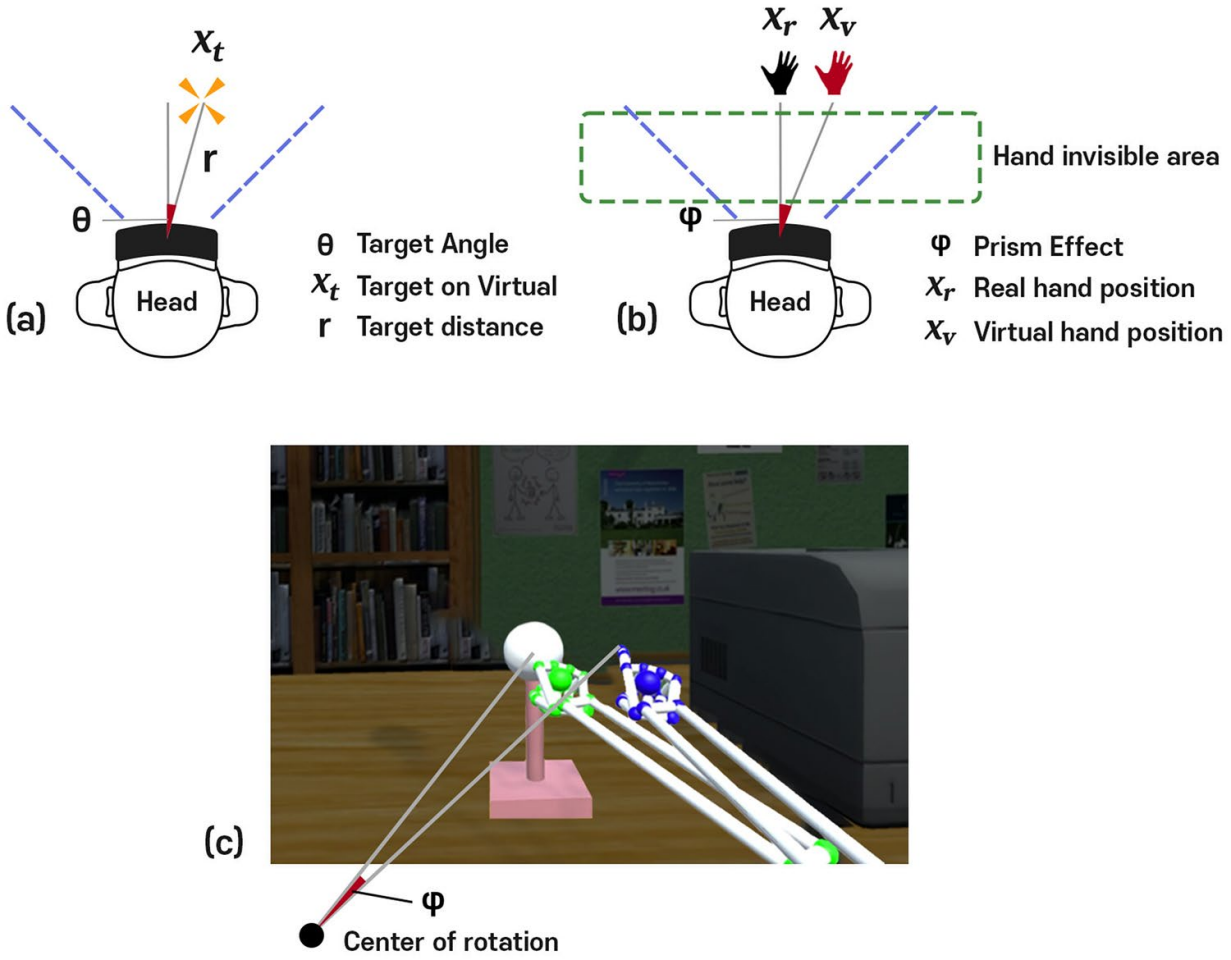
Hand trajectory deviations in VPAT system. (**a**) Target location ($${x}_{t})$$ defined as angle ($$\theta$$) and distance ($$r$$) from initial calibration, (**b**) Virtual hand position ($${x}_{v})$$ shifted to the right side at a deviation angle($$\varphi$$) from the real hand position ($${x}_{r})$$, (**c**) Hand trajectory: The green hand indicates the real hand trajectory and the blue hand indicates the virtual hand trajectory shifted to the right side.

The device was calibrated to determine where the target was located and visible. Figure 2c shows feedback that can only be seen when the index finger is within the visible area. The virtual hand is only visible beyond the invisible area shown in Fig. 3b. Even if the index finger does not exactly touch the target, the virtual hand becomes visible when the index finger reaches above the target's z distance. The target is a 6 cm diameter ball hanging from a rod. The virtual hand is not displayed until it is within a visible area, set to a depth similar to the target. When pointing at a target in a trial, the hand is prevented from being controlled accurately via real-time visual feedback.

The location of a target object on a virtual table is defined as angle $\uptheta$ from the center of view. In the initial calibration phase, the distance of the object is specified as a comfortably reachable distance allowing the user to touch them (Fig. 3a). First, the coordinate systems between the head and hand should be co-aligned. The coordinate system of the visual hand is transformed by the angle $\mathrm{\varphi }$ in relation to the head center (Fig. 3b). If a point from the HMD coordinate system is ${{\varvec{p}}}_{x\left|hmd\right.}$ and the point in the hand sensor coordinate system is ${{\varvec{p}}}_{x\left|hand\right.}$, the relationship is given by the following equation:

$$\left[\begin{array}{cc}{\varvec{R}}& {\varvec{d}}\\ 0& 1\end{array}\right]\left[\begin{array}{c}{{\varvec{p}}}_{x\left|hmd\right.}\\ 1\end{array}\right]=\left[\begin{array}{c}{{\varvec{p}}}_{x\left|hand\right.}\\ 1\end{array}\right]$$

where the matrix consisting of ***R*** and ***d*** is transformation matrix, representing the physical transformation between hand tracking sensor and HMD. The hand tracking sensor is attached to the front of the HMD without rotation. ***R*** is a 3 × 3 unit matrix where there is no rotation of sensor axes, and ***d*** is the relative position of the hand tracking sensor from the HMD. Second, the experimental condition is given by ${{\varvec{R}}}_{shift}$ and ${{\varvec{d}}}_{shift}$, which indicate rotation matrix and translation vector for hand shift, respectively. The hand shift was applied using the following equation:

$$\left[\begin{array}{cc}{{\varvec{R}}}_{shift}& {{\varvec{d}}}_{shift}\\ 0& 1\end{array}\right]\left[\begin{array}{cc}{\varvec{R}}& {\varvec{d}}\\ 0& 1\end{array}\right]\left[\begin{array}{c}{{\varvec{p}}}_{x\left|hmd\right.}\\ 1\end{array}\right]=\left[\begin{array}{c}{{\varvec{p}}}_{x\left|hand\right.}\\ 1\end{array}\right]$$

In our experiment, the hand only rotated horizontally. So, ${\mathbf{R}}_{shift}$ is

$$\begin{array}{ccc}{\cos}(\varphi )& 0& {\sin}(\varphi )\\ 0& 1& 0\\ -{\sin}(\varphi )& 0& {\cos}(\varphi )\end{array},$$

and $${{\varvec{d}}}_{shift}= 0$$.

### Link between the VPAT system and fNIRS

The VPAT system was connected to the fNIRS system, NIRScout (NIRx Medical Technologies, LLC, MN, USA). The VPAT signal acted as a trigger for recording in the fNIRS system. The VPAT system was implemented with Unity 3D. The remote keyboard control software using TCP/IP communication was also implemented to synchronize the starting event with the fNIRS system. The fNIRS system is controlled by Superlab 5.0 (Cedrus Corporation, San Pedro, USA, https://cedrus.com/superlab/) as an event trigger method. The remote command key in a computer where Superlab 5.0 is installed was used to initiate the fNIRS recording.

### Experimental design

**Figure 4:**
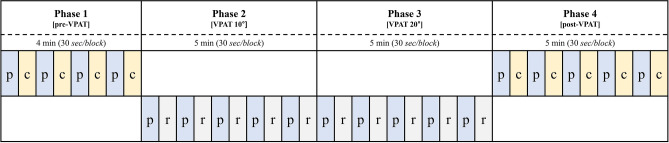
Experimental design. VPAT, virtual prism adaptation therapy; P pointing; C, clicking; R, resting.

Our protocol of video-recorded experiments was extensively documented previously^35^. Briefly, subjects sat comfortably and wore the VPAT system. They pointed the target in the VPAT system without the prism mode for simple familiarization where they experience target shifts in the prism mode. Then the fNIRS cap with optodes in the montage set for this study was added. The experimental design is shown in Fig. 4. The experiments started from Phase 1, no VPAT mode. Four pointing and four clicking blocks appeared alternatively for 4 min. Phase 2 and Phase 3 were conducted with VPAT mode (Phase 2: 10° deviation, Phase 3: 20° deviation); each phase was composed of five pointing blocks and five resting blocks, which appeared alternatively for 5 min. Phase 4 was conducted without VPAT mode and represents the post-prismatic adaptation period. Phase 4 consists of five pointing and clicking blocks alternatively appearing for 5 min. In the clicking block, subjects were instructed to click the button as soon as possible when the visual target appeared at 3 s intervals, using their right index finger. Each clicking block consisted of 10 clicks for 30 s. The pointing task block was composed of 10 pointing for 30 s in total. Subjects were instructed to point the visual targets with the right index finger as fast as possible. Clicking represents a task requiring attention but minimal motion, and pointing represents a task with motion and same level of attention as the clicking. Therefore, the cortical activation during pointing in contrast to clicking may reveal more activation on the left primary motor cortex. The visual target was presented on a position deviated 10° -right or 10°-left from the midline at 3-s intervals and in random orders. When the subject correctly pointed the target, the color changed to red.

### fNIRS measurement

**Figure 5:**
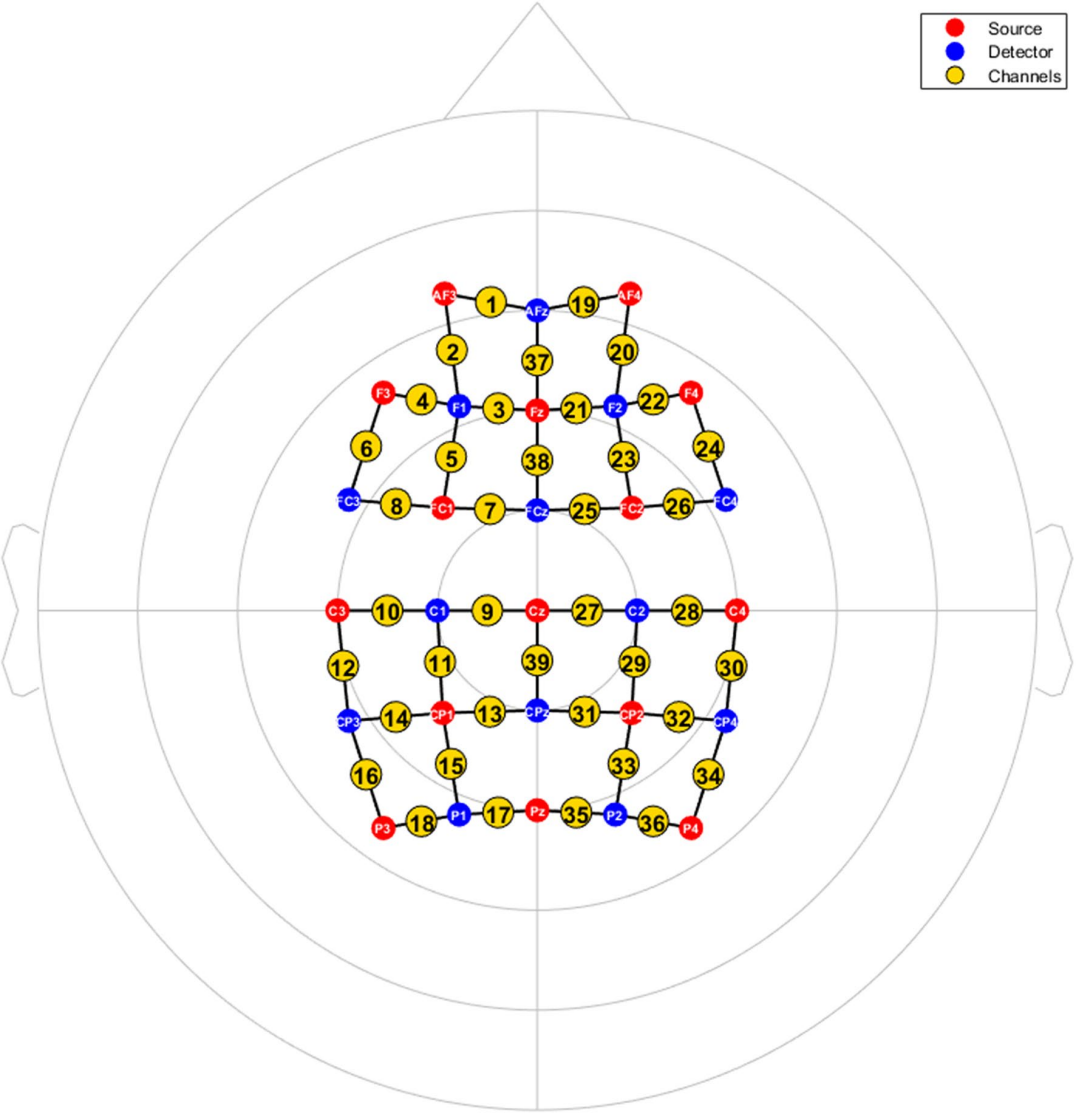
fNIRS recording montages for the experiment. Red circles indicate light sources, blue circles indicate detectors, and white letters designate the labels of the optodes, based on the 10–20 system. The yellow circles are the channels, which are numbered. The 2D coordinates of the optodes used in this figure were adopted from the NIRStar 14.1 program (NIRx Medical Technologies LLC, MN, USA, https://nirx.net/nirstar-1).

The continuous-wave type fNIRS system, NIRScout was used to measure changes in oxygenated hemoglobin [HbO] caused by cortical activations during the experiment. The manufacturer’s NIRStar 14.1 program was operated to control the NIRScout. The fNIRS system uses two wavelengths of near-infrared light, 760 and 850 nm, for measurement and records raw optical density data with a sampling rate of 4.17 Hz. A total of 39 measurement channels were formed by positioning 15 LED source and 13 detector optodes (optical probes). The optode positions were designed to mainly cover the dorsal frontoparietal network and the motor cortices of both hemispheres (Fig. 5), based on the international 10–20 system^36^. The separation between each source and detector was approximately 3 cm, enabling the near-infrared light to reach cortical areas. For optode fixation, textile EEG caps (EASYCAP, Herrsching, Germany) in three different sizes (54, 56, and 58 cm circumference) were prepared and selected depending on subject’s head size. To ensure the correct positioning of the applied optodes during the experiment, three researchers attended and checked the positions of four anatomical markers (Nasion, Inion, Left and right pre-auricular points (PAL and PAR respectively)) before and after the experiment for each subject.

### Pointing error analysis

All hand trajectory data was recorded with a sampling rate of 60 Hz during the experiment. The pointing error was calculated from the initial calibration position of HMD on the xz plane to the angle between the virtual hand pointing endpoint and the target position. The first pointing errors during each block in the four phases were used for analysis. They are presented using a box and whisker plot. Multiple Wilcoxon signed-rank tests were used to test the differences between six time points (three transitions between phases and three time points between the first and last block in phases 2 to 4). A 2-tailed Bonferroni correction, with a *p* < 0.008 considered statistically significant, was performed to adjust the type I error due to multiple comparisons. Statistical analyses were performed using the PASW statistical package (SPSS version 18.0, SPSS, https://www.ibm.com/analytics/spss-statistics-software).

### fNIRS data processing and analysis

Preprocessing was performed using the nirsLAB program, version 2019.04 (NIRx Medical Technologies LLC, MN, USA, https://nirx.net/nirslab-1)^37^. First, the optical density signal intervals contaminated with a motion artifact, drift, or spike, were visually inspected and manually corrected with the functions, ‘Remove Discontinuities’ and ‘Remove Spike artifacts’ of the program. The resultant optical density signals were bandpass filtered with a passband between 0.009 and 0.2 Hz to remove drifts and task-unrelated physiological effects, such as respiration and cardiac activities^38^. Then, the optical density raw data was converted to [HbO] by applying the modified Beer–Lambert Law^39^ with differential path lengths of 7.25 and 7.83, for the wavelengths 760 and 850 nm, respectively. The molar extinction coefficients (M^−1^ cm^−1^) were set to 1486.5865 for 760 nm and 2526.391 for 850 nm for HbO. [HbO] levels were used for analysis because it is more sensitive to the cortical blood flow changes according to the task and deoxyhemoglobin has shown considerable individual differences in task-related changes^40^.

General linear model (GLM)-based statistical data analysis for the task “pointing” was performed. In Level-1 analysis (within-subject), the GLM coefficients of [HbO] in each channel were estimated with the canonical hemodynamic response function (HRF) after temporal filtering by discrete cosine transform function and, in turn, precoloring by HRF was implemented. Then, the three t-contrasts were specified for the comparisons of the pointing blocks in each of the three phases (VPAT-10°, VPAT-20°, Post-VPAT) against the pointing blocks in the default phase, pre-VPAT. Subsequently, for each subject, SPM t-maps were computed based on those t-contrasts with p < 0.05 (uncorrected). In Level-2 analysis (group), group t-maps for the three comparisons were generated with t-tests to identify the channels most significantly activated in both positive and negative directions by pointing, in contrast to pre-VPAT pointing (VPAT-10° vs. Pre-VPAT, VPAT-20° vs. Pre-VPAT, Post-VPAT vs. Pre-VPAT). The most positive channel indicates the area most significantly activated by a specific phase against Pre-VPAT and the most negative channel indicates the most significant deactivation. All procedures were performed with Statistical Parametric Mapping (SPM, version 8, https://www.fil.ion.ucl.ac.uk/spm/software/spm8/)^41^, integrated within the nirsLAB program.

### Ethics approval and consent to participate

Written, informed consent was obtained from each subject. The Seoul National University Bundang Hospital Institutional Review Board (IRB) approved this study protocol (IRB number: B-1605/345-006).

## Results

### Pointing errors during the experiment

**Figure 6:**
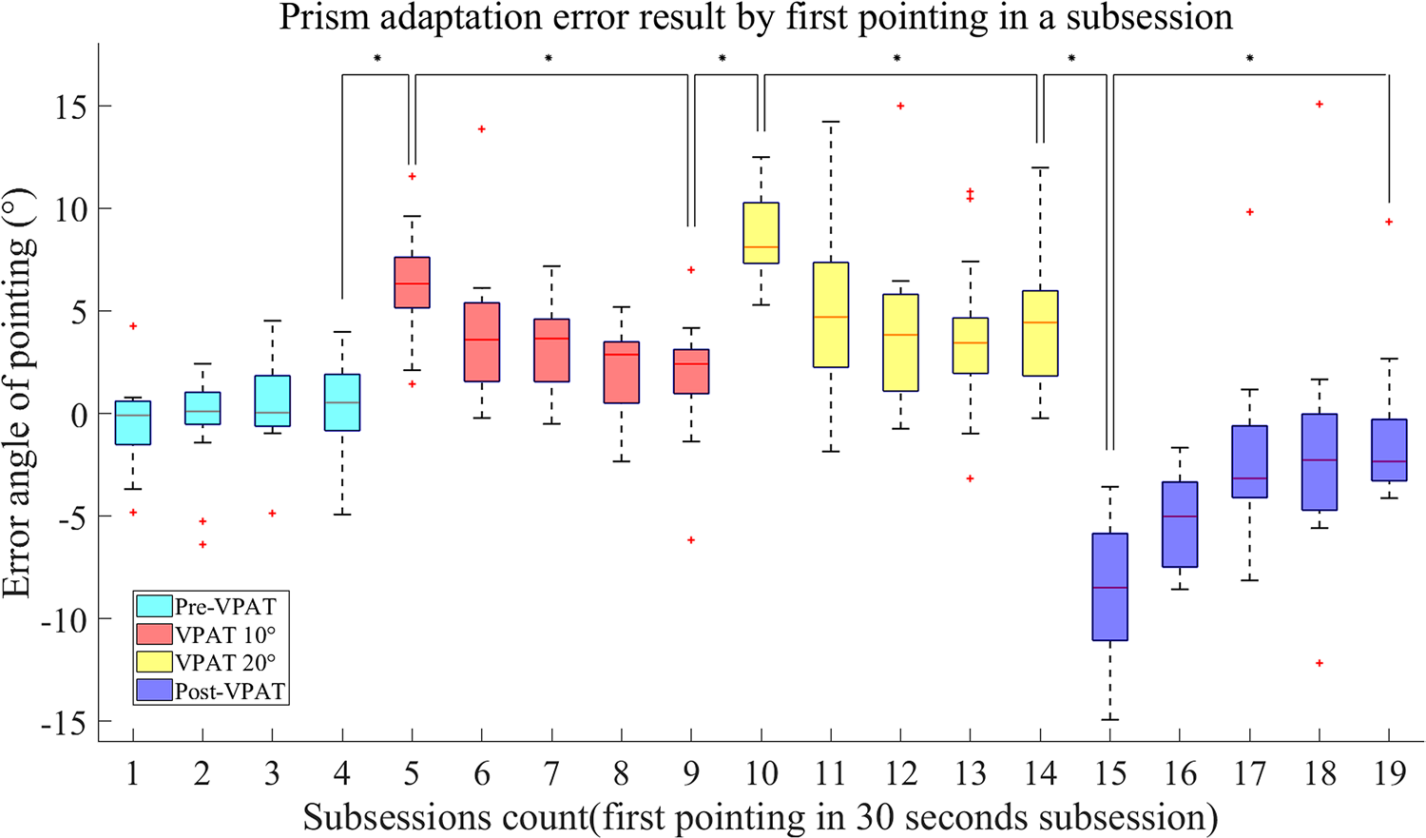
Pointing errors during the experiment. Positive value indicates rightward deviation, whereas negative value indicates leftward deviation. VPAT, virtual prism adaptation therapy. *p < 0.008 by Wilcoxon signed rank test with Bonferroni correction.

Pointing errors in each block were presented using a box and whisker plot (Fig. 6). Pointing errors were around 0° in the pre-VPAT phase. In the VPAT-10° phase, the errors initially deviated towards the right with a median value of 6.32° and interquartile range (IQR) of 3.00° and gradually decreased to 2.41° (IQR: 2.33°). At the start of the VPAT-20° phase, pointing errors moved rightward 8.10° (IQR: 3.00°) and decreased to 4.43° (IQR: 4.23°). In the post-VPAT phase, there was a substantial leftward pointing error (8.48°, IQR: 5.69°) in the first block of pointing, decreasing gradually afterward. The differences in pointing errors between the three phase transitions were statistically significant (*p* < 0.008), as were the changes between the first and last block in the three phases (VPAT-10°, 20° and post-VPAT phase) (*p* < 0.008) (Fig. 6). The average number of outliers was 0.83 (Standard deviation—1.40) in each block, and these were excluded from our analysis. The common causes of outliers were failure to point within the time frame, not returning the hand to the starting position before starting the next trial, or an error in hand tracking by Leap Motion camera.

### Cortical activation as measured by fNIRS

**Figure 7:**
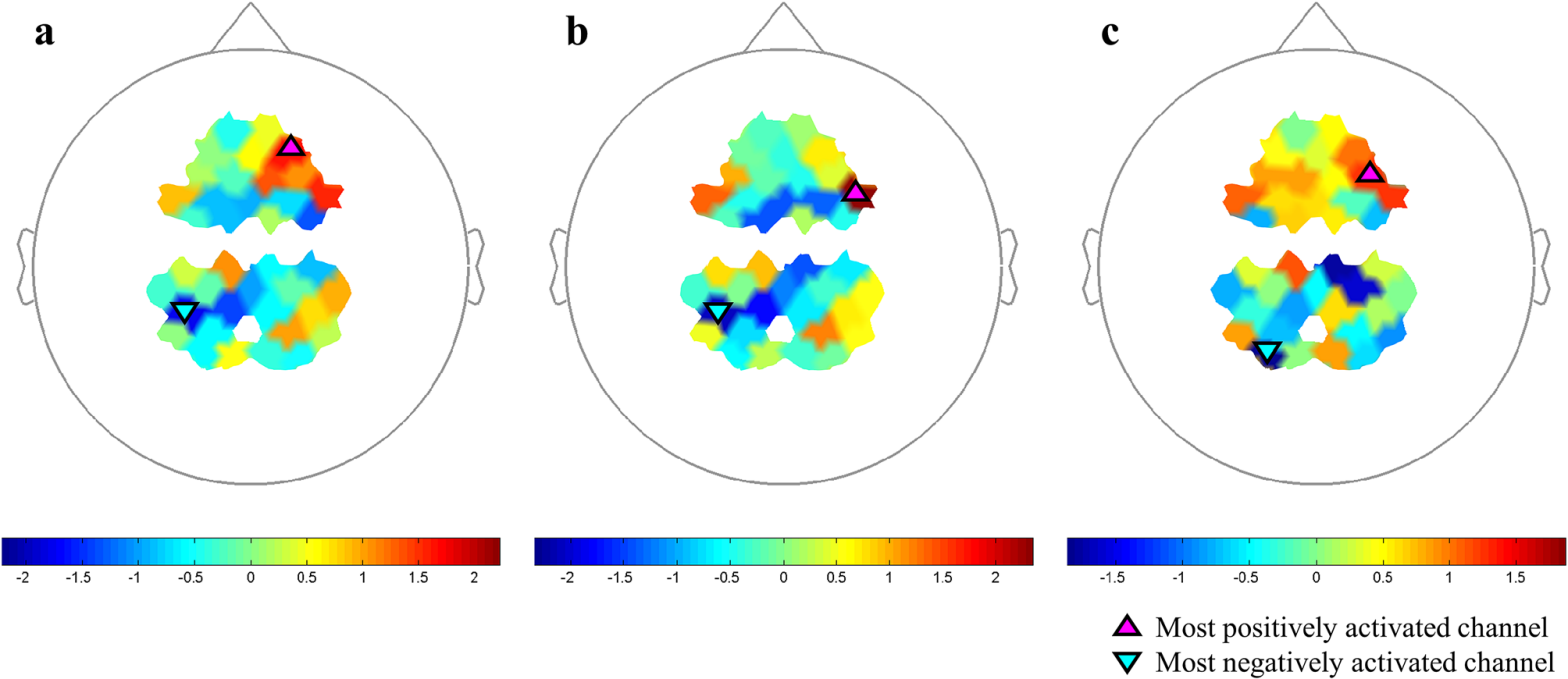
Cortical regions most activated during pointing in VPAT-10° (**a**), VPAT-20° (**b**) and post-VPAT (**c**) by contrast to the pre-VPAT pointing. The most significantly activated areas on the t-maps are marked with black-lined triangle symbols: pink-colored triangles for most positively activated areas and sky-blue-colored inverted triangles for most negatively activated areas. On each SPM t-map, each colored area corresponds to a single channel. Redder color means more activation during each phase in contrast to Pre-VPAT phase, and vice versa. VPAT, virtual prism adaptation therapy.

The cortical areas most significantly activated during pointing were identified by comparison to pointing trials during pre-VPAT in the following three phases: VPAT-10° (channel 20, *p* = 0.108), VPAT-20° (channel 24, *p* = 0.027) and post-VPAT (channel 22, *p* = 0.221) (Table 1 and Fig. 7). The most significantly activated channels were all located in the right hemisphere, and possible corresponding cortical areas include the dorsolateral prefrontal cortex and the frontal eye field (Brodmann areas 8, 9 and 46)^42^. The most deactivated channels were located around the left intraparietal sulcus. The cortical activation maps during pointing in each phase are presented in the supplementary Fig. S1.

**Table 1:** Cortical regions most significantly activated during virtual prism adaptation therapy (VPAT).

Condition	Positive activation				Negative activation			
	Side	Ch. no.	t	*p*	Side	Ch. no.	t	*p*
(VPAT-10° pointing)—(pre-VPAT pointing)	Right	20	1.663	0.108	Left	14	− 1.7813	0.0865
(VPAT-20° pointing)—(pre-VPAT pointing)	Right	24	2.342	0.027	Left	14	− 2.0637	0.0492
(Post-VPAT pointing)—(pre-VPAT pointing)	Right	22	1.254	0.221	Left	18	− 1.8661	0.0734

## Discussion

The aim of this study was to examine the feasibility of VPAT by assessing after-effect and cortical activation measured with fNIRS. After-effect, as measured with pointing errors, exhibited a similar response to conventional PA as reported in past papers. The most significantly activated channels determined by fNIRS during VPAT were right dorsolateral prefrontal cortex and the frontal eye field (Brodmann areas 8, 9 and 46), while the most deactivated channels were located around the left intraparietal sulcus.

The mechanisms used in our VPAT system differ from real prism therapy, in that in conventional PA, the whole visual field is shifted to the right. However, in our VPAT system, only the virtual hand trajectory is deviated to the right, while the actual target remains in its real position (Fig. 3)^43^. This difference in mechanism occurs due to the nature of immersive-VR and was found in the previous studies on VPAT using HMD^27,29,30^. According to Redding and Wallace^14^ realignment during PA occurs in an additive manner regarding visual and proprioceptive realignment, which is mainly responsible for after-effect from PA^13^. Newport et al.^44^ separated visual and proprioceptive realignment using real prism therapy and found that proprioceptive realignment alone was able to induce after-effect. On this basis, we hypothesized that VPAT, which induces proprioceptive realignment, can generate after-effect.

The open-loop pointing errors in post-VPAT adaptation at 20° deviation was 8.48° in our study, which is similar to a previous study performed in healthy individuals^27^. Whether the size of after-effect induced by VPAT is comparable to the conventional PA remains controversial. The after-effect of conventional PA reported by Newport et al.^44^ and Michel et al.^45^ were larger, while Ramos et al.^28^ compared conventional PA with two types of VPAT and demonstrated that VPAT can generate a larger after-effect than conventional PA. Moreover, after-effect measured with open-loop pointing error is considered to be strongly related to transfer^15^; however, findings on the relationship between after-effect and transfer using VPAT in healthy individuals^27,28^ and stroke patients with USN^30^ were not consistent with findings in conventional PA. Gammeri et al. demonstrated a dose–response effect of VPAT as only participants adapted to 30° showed transfer^27^, while Bourgeois et al. have shown no transfer in the patients with USN although after-effect was observed^30^. Since our study did not investigate transfer, we could not conclude whether after-effect induced by VPAT can lead to transfer. These findings raise the question of whether the quality of after-effect and underlying neural mechanism of VPAT is comparable to that of conventional PA, and further study is required to elucidate the relationship between after-effect and transfer in VPAT.

Investigation of the relevant cortical area related to VPAT was another objective in this study. Since the induction mechanism of behavioral adaptation in our VPAT may be different from conventional therapy using a real prism, comparison of the activated cortical areas with previous studies on cortical activations with a real prism should be scrutinized. Although no other studies have used fNIRS to assess cortical activation during conventional PA, studies using fMRI^34,46,47^ have shown that both parietal cortex and cerebellum are activated during the recalibration and realignment process in PA, while the cognitive effect of PA by bottom-up activation occurs in temporo-occipital and prefrontal areas^34,48^. In our study, the most activated areas during and after VPAT included the right frontal eye field and the dorsolateral prefrontal cortex (Table 1 and Fig. 7)^42^, although without statistical significance in the VPAT-10° and post-VPAT periods.

The frontal eye field is an area involved in the dorsal attentional network, which mediates top-down stimulus–response selection^49^, producing inattention to an object in the contralesional far-space when that area is damaged^50^. A recent functional MRI study found that rightward PA increased resting functional connectivity in healthy adults between the right frontal eye field and right anterior cingulate cortex after PA, which may mean more attention to the left visual field^51^.

In our study, the activated area was the right dorsolateral prefrontal cortex, not the left. A previous functional MRI study in patients with right hemispheric damage showed that rightward PA increased activation in the left prefrontal cortex^48^. It was postulated that PA induced bottom-up activation of the prefrontal cortex, which then led to the enhanced function of the left hemisphere to perceive the left visual field^16^. However, the role of the left prefrontal cortex on PA’s effect has not been well elucidated. Bottom-up stimulus driven control is known to be related to the ventral attentional network usually including the ventral prefrontal cortex, not the dorsal one^52^. In addition, despite an ongoing debates, the dorsolateral prefrontal cortex is considered to be included in the dorsal attentional network^52,53^. The shift from using the left ventral attentional network by PA, secondarily activates the right dorsal attentional network to perceive the left visual field, thus in our study, VPAT may induce the activation of the dorsal attentional network including both the right dorsolateral prefrontal cortex and frontal eye field. The various subject characteristics (right prefrontal cortex damaged patients vs. healthy adults) could also result in the different activation side in the prefrontal cortex between our study and that by Croattaz-Herbette et al.^48^. Due to these uncertainties in the role of the prefrontal cortex on PA and the difficulties in directly measuring the hemodynamic changes of the deeply located ventral prefrontal cortex and cerebellum by fNIRS, further research is warranted.

Application of immersive VR to PA has several advantages compared to conventional PA: (1) gradual adjustment of deviations according to the patient’s adaptation^27^, (2) easier adjustment of deviation angles^28^, (3) possibility of combination with other therapeutic devices^29^, (4) blockage of external visual cues, and (5) quantification and monitoring of pointing errors during therapy. The VPAT system can help patients and therapists set up treatment environments in the patient’s home, with monitoring or self-guided algorithms (e.g., automatically adjusting the deviations according to the patient’s behavioral adaptation). Despite these possible advantages of VPAT, further study on VPAT is still required as the neural mechanism of adaptation in VPAT has not yet been elucidated. Moreover, evaluation of the transfer effect of VPAT on visuo-motor cognitive domain and alleviation of USN needs to be performed to establish VPAT as an alternative therapeutic tool to conventional PA in the treatment of USN in stroke patients.

This study has several limitations. First, the study did not assess the transfer of adaption effect. Considering that the purpose of PA is to ameliorate USN, transfer to various visuo-motor cognitive domains needs to be examined in future studies. Second, the exact localization of involved cortical areas using fNIRS was difficult without co-registration of each channel with an individual magnetic resonance image (MRI) or mapping to a normalized brain based on the digitization of anatomical markers. We attempted to alleviate this limitation by following the 10–20 system, using an appropriately sized cap according to the subject’s head size and identifying channels able to measure areas of interest by applying the fNIRS optodes’ location decider program^42^. In addition, deeply located structures possibly related with the effect of PA such as the cerebellum^34,46^ and anterior cingulate cortex^54^ could not be directly monitored by fNIRS, which hindered us from investigating the cortical network underlying VPAT effect, such as the cerebello-parietal network^16^. Third, the contamination influence from extracerebral layers, such as Mayer's wave, was not eliminated. This complicated the interpretation of the results. With the current fNIRS system with a source-detector separation of 3 cm, global regression was a recommended option. However, this could not be applied because the unequal weight distribution and the fixation band of the VPAT device might violate the assumption that the extracerebral contamination was applied equally across all channels. Fourth, the single statistically activated channel was only found in the VPAT-20° condition, although most significantly activated channels were consistently located around the right dorsolateral prefrontal cortex and frontal eye field. This can be explained by VPAT’s dose–response effect as previously shown by Gammeri et al.^27^ In addition, a total of 50 pointing with 30 s resting time after each 10 pointing were used in our experiment, which was lower than the number of consecutive pointing (one hundred) in previous VPAT studies^27,30^. Finally, in situations where the hand was not detected properly due to occlusions or self-occlusions, there were often cases where the hand did not appear, even though it actually touched the virtual object during VPAT. Using VR controllers would reduce this error. However, fatigue might occur due to the holding pose and the controller weight. Hand tracking technology using multiple cameras on the front of the HMD could reduce errors caused by occlusion without fatigue^55^.

## Conclusions

The VPAT system, which deviated the hand trajectory rightward induced after-effect. The most activated cortical areas during and immediately after VPAT were the dorsolateral prefrontal cortex and the frontal eye field, which are associated with the dorsal attentional network. Our findings demonstrated the feasibility of VPAT in producing after-effect and identified possible neural networks activated during the adaptation process, However, whether the underlying neural mechanism of VPAT and its therapeutic effect are comparable to that of conventional PA still requires further scrutiny. Future clinical trials using VPAT with a high degree of rightward deviation with multiple sessions over longer periods, assessment of transfer to visuo-motor cognitive domain, and comparison with conventional PA is required in stroke patients with USN.

## Supplementary Information

Supplementary Information 1.

Supplementary Information 2.

Supplementary Information 3.

## Data Availability

The datasets generated and analyzed during the current study are available on the supplementary data.

## Abbreviations

USN: Unilateral spatial neglect
PA: Prism adaptation
VPAT: Virtual prism adaptation therapy
VR: Virtual reality
fNIRS: Functional near-infrared spectroscopy
HMD: Head mounted display
GLM: General linear model
HRF: Hemodynamic response function
IQR: Interquartile range

## References

[1] Feigin VL (2014). Global and regional burden of stroke during 1990–2010: Findings from the Global Burden of Disease Study 2010. Lancet.

[2] Lawrence ES (2001). Estimates of the prevalence of acute stroke impairments and disability in a multiethnic population. Stroke.

[3] Brewer, L., Horgan, F., Hickey, A. & Williams, D. Stroke rehabilitation: recent advances and future therapies. *Qjm*, **106**(1):11–25 hcs174 (2013). 10.1093/qjmed/hcs174 23019591

[4] Appelros P, Karlsson GM, Seiger A, Nydevik I (2002). Neglect and anosognosia after first-ever stroke: Incidence and relationship to disability. J. Rehabil. Med..

[5] Buxbaum L (2004). Hemispatial neglect subtypes, neuroanatomy, and disability. Neurology.

[6] Jehkonen M (2000). Visual neglect as a predictor of functional outcome one year after stroke. Acta Neurol. Scand..

[7] Jehkonen M, Laihosalo M, Kettunen J (2006). Impact of neglect on functional outcome after stroke—A review of methodological issues and recent research findings. Restor. Neurol. Neurosci..

[8] Saevarsson S, Halsband U, Kristjánsson Á (2011). Designing rehabilitation programs for neglect: Could 2 be more than 1+ 1?. Appl. Neuropsychol..

[9] Kerkhoff G, Schenk T (2012). Rehabilitation of neglect: An update. Neuropsychologia.

[10] Yang NY, Zhou D, Chung RC, Li-Tsang CW, Fong KN (2013). Rehabilitation interventions for unilateral neglect after stroke: A systematic review from 1997 through 2012. Front. Hum. Neurosci..

[11] Barrett A, Goedert KM, Basso JC (2012). Prism adaptation for spatial neglect after stroke: Translational practice gaps. Nat. Rev. Neurol..

[12] Prablanc C (2020). Adapting terminology: Clarifying prism adaptation vocabulary, concepts, and methods. Neurosci. Res..

[13] Petitet P, O’Reilly JX, O’Shea J (2018). Towards a neuro-computational account of prism adaptation. Neuropsychologia.

[14] Redding GM, Wallace B (2006). Generalization of prism adaptation. J. Exp. Psychol. Hum. Percept. Perform..

[15] Redding GM, Wallace B (2006). Prism adaptation and unilateral neglect: Review and analysis. Neuropsychologia.

[16] Panico F, Rossetti Y, Trojano L (2020). On the mechanisms underlying Prism Adaptation: A review of neuro-imaging and neuro-stimulation studies. Cortex.

[17] Serino A, Bonifazi S, Pierfederici L, Làdavas E (2007). Neglect treatment by prism adaptation: What recovers and for how long. Neuropsychol. Rehabil..

[18] Serino A, Barbiani M, Rinaldesi ML, Làdavas E (2009). Effectiveness of prism adaptation in neglect rehabilitation. Stroke.

[19] Mizuno K (2011). Prism adaptation therapy enhances rehabilitation of stroke patients with unilateral spatial neglect: A randomized, controlled trial. Neurorehabil. Neural Repair.

[20] Shiraishi H, Yamakawa Y, Itou A, Muraki T, Asada T (2008). Long-term effects of prism adaptation on chronic neglect after stroke. NeuroRehabilitation.

[21] Frassinetti F, Angeli V, Meneghello F, Avanzi S, Làdavas E (2002). Long-lasting amelioration of visuospatial neglect by prism adaptation. Brain.

[22] Rusconi ML, Carelli L (2012). Long-term efficacy of prism adaptation on spatial neglect: Preliminary results on different spatial components. Sci. World J..

[23] Ten Brink AF (2017). Prism adaptation in rehabilitation? No additional effects of prism adaptation on neglect recovery in the subacute phase poststroke: A randomized controlled trial. Neurorehabil. Neural Repair.

[24] Nys GM, de Haan EH, Kunneman A, de Kort PL, Dijkerman HC (2008). Acute neglect rehabilitation using repetitive prism adaptation: A randomized placebo-controlled trial. Restor. Neurol. Neurosci..

[25] Maxton C, Dineen R, Padamsey R, Munshi S (2013). Don’t neglect ‘neglect’—An update on post stroke neglect. Int. J. Clin. Pract..

[26] Newport R, Schenk T (2012). Prisms and neglect: What have we learned?. Neuropsychologia.

[27] Gammeri R, Turri F, Ricci R, Ptak R (2020). Adaptation to virtual prisms and its relevance for neglect rehabilitation: A single-blind dose-response study with healthy participants. Neuropsychol. Rehabil..

[28] Ramos AA, Hørning EC, Wilms IL (2019). Simulated prism exposure in immersed virtual reality produces larger prismatic after-effects than standard prism exposure in healthy subjects. PLoS ONE.

[29] Wilf M (2021). Combined virtual reality and haptic robotics induce space and movement invariant sensorimotor adaptation. Neuropsychologia.

[30] Bourgeois A, Turri F, Schnider A, Ptak R (2021). Virtual prism adaptation for spatial neglect: A double-blind study. Neuropsychol. Rehabil..

[31] Yang M, Yang Z, Yuan T, Feng W, Wang P (2019). A systemic review of functional near-infrared spectroscopy for stroke: Current application and future directions. Front. Neurol..

[32] Quaresima V, Ferrari M (2019). Functional near-infrared spectroscopy (fNIRS) for assessing cerebral cortex function during human behavior in natural/social situations: A concise review. Organ. Res. Methods.

[33] Caplan B, Mendoza JE (2011). Encyclopedia of Clinical Neuropsychology.

[34] Luauté J (2009). Dynamic changes in brain activity during prism adaptation. J. Neurosci..

[35] Cho S, Kim WS, Park SH, Park J, Paik NJ (2020). Virtual prism adaptation therapy: Protocol for validation in healthy adults. J. Vis. Exp..

[36] Klem GH, Luders HO, Jasper HH, Elger C (1999). The ten-twenty electrode system of the International Federation. The International Federation of Clinical Neurophysiology. Electroencephalogr. Clin. Neurophysiol..

[37] Xu, Y., Graber, H. L. & Barbour, R. L. In *Biomedical Optics.* BM3A. 1 (Optical Society of America).

[38] Tachtsidis I, Scholkmann F (2016). False positives and false negatives in functional near-infrared spectroscopy: Issues, challenges, and the way forward. Neurophotonics.

[39] Delpy DT (1988). Estimation of optical pathlength through tissue from direct time of flight measurement. Phys. Med. Biol..

[40] Strangman G, Franceschini MA, Boas DA (2003). Factors affecting the accuracy of near-infrared spectroscopy concentration calculations for focal changes in oxygenation parameters. Neuroimage.

[41] Penny WD, Friston KJ, Ashburner JT, Kiebel SJ, Nichols TE (2011). Statistical Parametric Mapping: The Analysis of Functional Brain Images.

[42] Zimeo Morais GA, Balardin JB, Sato JR (2018). fNIRS Optodes' Location Decider (fOLD): A toolbox for probe arrangement guided by brain regions-of-interest. Sci. Rep..

[43] Aasted CM (2015). Anatomical guidance for functional near-infrared spectroscopy: AtlasViewer tutorial. Neurophotonics.

[44] Newport R, Preston C, Pearce R, Holton R (2009). Eye rotation does not contribute to shifts in subjective straight ahead: Implications for prism adaptation and neglect. Neuropsychologia.

[45] Michel C, Cruz R (2015). Prism adaptation power on spatial cognition: Adaptation to different optical deviations in healthy individuals. Neurosci. Lett..

[46] Küper M (2014). Activation of the cerebellar cortex and the dentate nucleus in a prism adaptation fMRI study. Hum. Brain Mapp..

[47] Chapman HL (2010). Neural mechanisms underlying spatial realignment during adaptation to optical wedge prisms. Neuropsychologia.

[48] Crottaz-Herbette S (2017). Reshaping the brain after stroke: The effect of prismatic adaptation in patients with right brain damage. Neuropsychologia.

[49] Corbetta M, Kincade MJ, Lewis C, Snyder AZ, Sapir A (2005). Neural basis and recovery of spatial attention deficits in spatial neglect. Nat. Neurosci..

[50] Rizzolatti, G., Berti, A. & Gallese, V. *Spatial Neglect: Neurophysiological Bases, Cortical Circuits and Theories* (2000).

[51] Tsujimoto K (2019). Prism adaptation changes resting-state functional connectivity in the dorsal stream of visual attention networks in healthy adults: A fMRI study. Cortex.

[52] Corbetta M, Shulman GL (2002). Control of goal-directed and stimulus-driven attention in the brain. Nat. Rev. Neurosci..

[53] Qian S (2020). Disrupted anti-correlation between the default and dorsal attention networks during hyperthermia exposure: An fMRI study. Front. Hum. Neurosci..

[54] Danckert J, Ferber S, Goodale MA (2008). Direct effects of prismatic lenses on visuomotor control: An event-related functional MRI study. Eur. J. Neurosci..

[55] Han S (2020). MEgATrack: Monochrome egocentric articulated hand-tracking for virtual reality. ACM Trans. Graph..

