# Supplementary material for: Feasibility study of immersive virtual prism adaptation therapy with depth-sensing camera using functional near-infrared spectroscopy in healthy adults
Source: Sci Rep. 2022 Jan 14;12:767. doi: 10.1038/s41598-022-04771-5 (PMC8760318; doi:10.1038/s41598-022-04771-5)
Supplement: Supplementary file 1 — Supplementary Information 1. [file 41598_2022_4771_MOESM1_ESM.docx]

**Feasibility study of immersive virtual prism adaptation therapy with depth-sensing camera using functional near-infrared spectroscopy in healthy adults**

Sungmin Cho^1#^, Won Kee Chang^1#^, Jihong Park^1^, Seung Hyun Lee^2^, Jongseung Lee^1^, Cheol E. Han^3,4^, Nam-Jong Paik^1^, Won-Seok Kim^1*^

^1^Department of Rehabilitation Medicine, Seoul National University College of Medicine, Seoul National University Bundang Hospital, Seongnam, Republic of Korea

^2^Global Health Technology Research Center, College of Health Science, Korea University, Seoul, Republic of Korea

^3^Department of Electronics and Information Engineering, Korea University, Sejong, Republic of Korea

^4^ Interdisciplinary Graduate Program for Artificial Intelligence Smart Convergence Technology, Korea University, Sejong, Republic of Korea


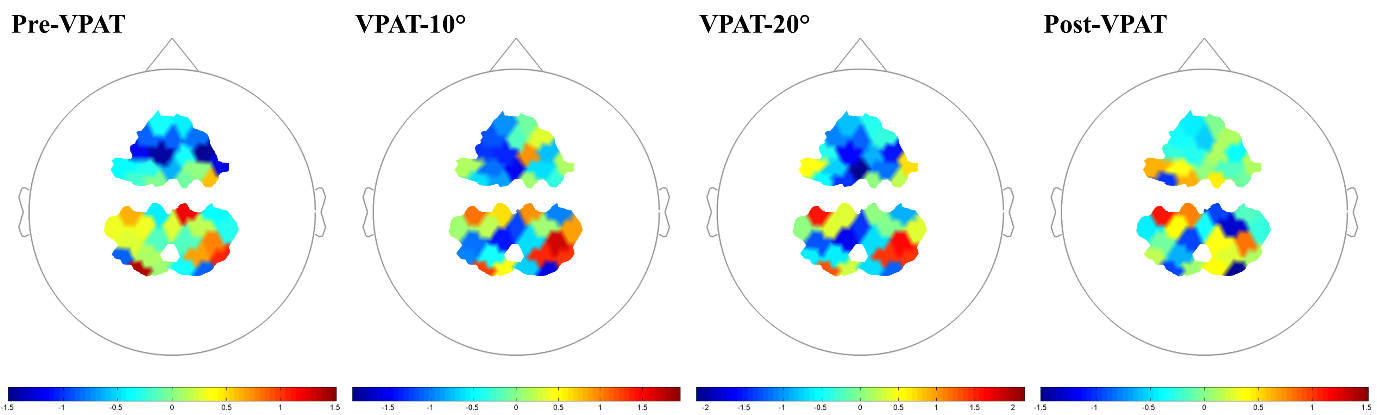


**Supplementary Figure S1.** **Cortical activation maps during pointing in each phase.** On each SPM t-map, each colored area corresponds to a single channel. Redder color means more activation during each phase in contrast to Pre-VPAT phase, and vice versa. VPAT, virtual prism adaptation therapy.

**Supplementary Data 1. Raw data for pointing error analysis in each block of each phase.**

**Supplementary Data 2. Raw data for fNIRS data analysis.**
